# The Effect of Age, Gender and Comorbidities Upon SARS-CoV-2 Spike Antibody Induction After Two Doses of Sinopharm Vaccine and the Effect of a Pfizer/BioNtech Booster Vaccine

**DOI:** 10.3389/fimmu.2022.817597

**Published:** 2022-05-30

**Authors:** Eman Farid, Juber Herrera-Uribe, Nigel J. Stevenson

**Affiliations:** ^1^ Department of Microbiology, Immunology and Infectious Diseases, College of Medicine, Arabian Gulf University, Manama, Bahrain; ^2^ Department of Pathology, Salmanyia Medical Complex, Government Hospital, Manama, Bahrain; ^3^ Laboratory Department, Al Salam Specialist Hospital, Riffa, Bahrain; ^4^ Viral Immunology Group, School of Biochemistry and Immunology, Trinity Biomedical Sciences Institute, Trinity College Dublin, Dublin, Ireland; ^5^ Viral Immunology Group, Royal College of Surgeons in Ireland (RCSI), Medical College of Bahrain, Busaiteen, Bahrain

**Keywords:** vaccination, antibody quantification, SARS-CoV-2, spike protein, Sinopharm, Pfizer/BioNtech

## Abstract

Severe acute respiratory syndrome coronavirus (SARS-CoV)-2 emerged in China in 2019 and has since travelled the world infecting millions. SARS-CoV-2 causes Corona Virus Disease (COVID-19), that has to date taken over 4 million lives. The Kingdom of Bahrain’s vaccine roll-out has consisted of Sinopharm’s BBIBP-CorV (Sinopharm) and Pfizer/BioNtech’s BNT162b2 (Pfizer/BioNtech). Testing for SARS-CoV-2 anti-Spike (S) antibodies is a useful technique in estimating an individual’s immune protection against the infection. In this study we evaluated S antibody levels by electro-chemiluminescence immunoassay in 379 individuals double vaccinated with Sinopharm and 15 of whom were given a booster with the Pfizer/BioNtech vaccine. Among our double vaccinated cohort, we found a spectrum of S antibody levels. Indeed, we found that a significant proportion of individuals with low S antibody levels had clinical conditions, which were mainly immune-related disorders. Furthermore, a significant proportion of individuals with low S antibody levels were above 50 years of age. Finally, we observed a significant increase in S antibody levels after the Pfizer/BioNtech booster was administered. These findings reveal that while a large proportion of Sinopharm vaccinated individuals did not develop high levels of antibodies against the S protein, a booster dose of the Pfizer/BioNtech vaccine significantly enhances S antibody levels, revealing this “triple dose” vaccination strategy as a useful method of ensuring protective immunity against SARS-CoV-2.

## Introduction

Severe acute respiratory syndrome coronavirus 2 (SARS-CoV-2) causes Corona Virus Disease (COVID)-19. The virus emerged in Wuhan, China in 2019, but its global spread was quickly declared a pandemic by the World Health Organization (WHO), as it rapidly infected millions of humans worldwide. COVID-19 has caused more than 1.4 million deaths as of 22 July 2021 (1). SARS-CoV-2 is a single, positive-strand RNA virus that can cause respiratory, gastrointestinal and central nervous system disease, that is readily transmitted and spread in human and animals (2). Severe respiratory distress, pneumonia, renal failure and death are associated with severe forms of the infection (3). SARS-CoV-2 is spherical in form, with a diameter of 125 nm. The main feature of the SARS-CoV-2 and other CoVs is the raised club-shaped spike (S) protein on their surface. The virus uses its S protein to bind the cellular Angiotensin Converting Enzyme 2 (ACE2) receptor and subsequently infect the host cell (4). Although the SARS-CoV-2 genome encodes four structural proteins (S, envelope-E, membrane-M, nucleocapsid-N), sixteen non-structural proteins, and eight accessory proteins, the S protein is the most important for viral binding, fusion and entry into host cells (5).

Bahrain, a country with a population of ~1.641 million people, reported the first COVID-19 case in late February 2020, followed by an exponential growth of positive cases (6). The fast spread of SARS-CoV-2 infection around the world sparked a massive effort to create COVID-19 vaccinations, that could be globally administered to terminate the pandemic (7).

At the time of our study, there were seven vaccines approved for human use by the WHO. mRNA (Moderna mRNA-1273, Pfizer/BioiNTech BNT162b2), Non-Replicating Viral Vector (Janssen/Johnson & Johnson Ad26.COV2.S, Oxford/AstraZeneca AZD1222, Serum Institute of India Covishield) and inactivated (Sinovac CoronaVac, Sinopharm/Beijing BBIBP-CorV) vaccines have been used since 2020, for the immunization programs against the SARS-CoV-2, around the world^1^. The mRNA and viral vector vaccines have been developed to target the S protein, while the inactivated vaccines target the whole virion (7). Bahrain approved the use of the Sinopharm and Pfizer/BioNtech vaccines on the 14th of December 2020^2^. The inactivated Sinopharm vaccine, uses the whole virus, essentially retaining the immunogenicity, while lacking infectivity; these whole viruses are taken up by innate immune cells, digested and presented to adaptive immune cells, which, in turn, provide cellular and antibody-mediated protection (8). The Pfizer/BioNtech mRNA vaccine introduces the S gene into the host cell, which leads to the S protein being produced and presented to the adaptive immune cells, also generating cellular and antibody-mediated protection (4, 9).

According to John Hopkins University of Medicine, to date, Bahrain has reported 268,0992 positive SARS-CoV-2 cases and 1,381 related deaths. At the time of the study, 2,248,554 doses of vaccines were administered in Bahrain, with 1,036,747 people double vaccinated (63.17% of Bahraini population)^3^. As of August 2021, 174,436 of those who were given a double Sinopharm vaccination, have also been given a third “booster” dose of either the Sinopharm or Pfizer/BioNtech vaccines.

Although Bahrain’s vaccination program has covered more than 63% of the population, the virus, especially new variants, continue to infect even vaccinated individuals (10, 11), highlighting the need to analyze and monitor the level of antibodies after vaccination, especially in high-risk groups (people >50 years old and people with comorbidities) (12). Furthermore, the duration of vaccine-mediated protection and effect of a third “booster” dose remains poorly understood. Therefore, by analyzing S antibody levels in Sinopharm double vaccinated individuals, this study aims to shed light on the level of S antibodies induced by this vaccine, in both healthy and immune compromised individuals. Furthermore, by measuring the antibody levels of patients given a booster dose of the Pfizer/BioNtech vaccine, our study also aims to determine if third doses are useful in generating higher antibody levels. In summary, we have discovered a wide range of S antibody levels in our double Sinopharm vaccinated group and have found significantly lower S antibody levels in older people (above 50 years of age) and those with other clinical conditions. However, we also found that a booster of the Pfizer/BioNtech BNT162b2 vaccine significantly increases S antibody levels, revealing a vaccine strategy that ensures high levels of S antibody production.

## Material And Methods

### Study Design

A total of 379 Bahrain residents of both sexes, 18 years of age or older, who accepted to participate in this Sinopharm and Pfizer/BioNtech SARS-CoV-2 vaccination study between January 16 and July 2021 were under the care of Dr. Farid and thus included consecutively in this study. Bahrain residents who had previously contracted SARS-CoV-2 were not included in this study. This study was approved by the Al Salam Specialist Hospital management. All participants signed the voluntary informed consent form. Clinical data information was collected from all individuals involved in the study.

### Inactive BBIP-CorV (Sinopharm) and mRNA BNT162b2 (Pfizer/BioNtech) Vaccines

The Sinopharm vaccine is an inactivated virus, developed by the Beijing Institute of Biological Products (Beijing, China) (13). Sinopharm’s SARS-CoV-2 strain (WIV04 strain and GeneBank number MN996528) was isolated from a patient admitted to the Jinyintan Hospital, Wuhan, China. The SARS-CoV-2 strain was grown in kidney cell cultures (Vero cell line) of the African green monkey and was inactivated using β-propiolactone. A single dose of Sinopharm vaccine (0.5 mL) contains a harvested viral solution and an aluminum hydroxide adjuvant.

Pfizer-BioNTech’s vaccine is composed of nucleoside-modified mRNA that encodes a mutated form of the full-length SARS-CoV-2 S gene, which is encapsulated in lipid nanoparticles. The Pfizer/BioNtech vaccine is delivered in vials of dry powder, which is diluted in 0.9% Sodium Chloride sterile water for injection (USP; 2.25 mL).

### Vaccination Protocol

All participants involved in the study received two doses of the inactive BBIP-CorV vaccine (Sinopharm vaccine). Vaccination with Sinopharm vaccine was performed in the 27 government health centers distributed all over the country, with the recommended dosing interval of 21 days between the first and second doses, administered in the deltoid muscle^4^. Following the initial double Sinopharm vaccination, Bahrain offered a booster dose of either the Sinopharm or Pfizer/BioNtech vaccine, 3 months after the second dose of Sinopharm vaccine for certain groups (high risk category group [> 50 years, BMI > 40, immunocompromised and frontliners]) and 6 months after the second dose of Sinopharm vaccine for the rest of the population (low-risk groups). Only the Pfizer/BioNtech booster data was collected and reported in this study. 15 individuals from the initial cohort of 379 accepted to participate in the Pfizer/BioNtech booster analysis.

### Sample Collection

Blood samples were collected from individuals to determine the levels of SARS-CoV-2 S antibody. Blood donation was performed 14-31 days after the second dose of vaccination. Donors underwent standard venipuncture at the Al Salam Specialist Hospital. Serum was separated from blood after centrifugation and analysis was performed immediately.

### Analysis of Samples

Elecsys^®^ Anti-SARS-CoV-2 S (Roche) electro-chemiluminescence immunoassay for the *in vitro* quantitative determination of antibodies (including IgG) to the SARS-CoV-2 S protein receptor binding domain (RBD) in human serum was used in this study. Each sample (20 μL) was incubated with a mix of biotinylated and ruthenylated RBD antigen (double-antigen sandwich immune complex). Streptavidin-coated microparticles were added to the mix to capture formed complexes. Magnetic selection was applied to capture the immune complexes for further measurement in the Cobas e 411 analyzer (Roche).

### Interpretation of Results

The resulting electro-chemiluminescence reaction was measured as U/mL. The assay has an analytical linear range of 0.4 to 250 U/mL. Values <0.8 U/mL. were reported as non-reactive (negative) for SARS-CoV-2 spike (S) protein and values ≥0.8 U/mL were reported as reactive (positive). The assay has a specificity of 100% (95% confidence interval [95% CI]: 99.7%-100%).

### Statistical Analysis

A minimum sample size of 362 participants was confirmed to be appropriate using Raosoft Sample Size Calculator (Raosoft, Inc 2004); therefore, our sample size of 379 was sufficient. The sample size was considered appropriate according to the Bahrain population size (1.702 million), percentage of vaccinated Bahrain residents until July 2021, a margin error of 5% and confidence level of 95%. Descriptive statistical analysis was performed for continuous (median and minimum-maximum values) and qualitative variables (percentages). Mann-Whitney non-parametric test was used to compare the S antibody levels between genders, and between healthy donors and donors with other clinical conditions. For age groups comparisons, one way analysis non-parametric test (Kruskal Wallis test and Dunn’s multiple comparison) was performed. The adequate sample size for each age group comparison was calculated using EpiTools^5^. Assumptions included mean and variance for each age group, confidence level (0.95), power (0.8), ratio of sample sizes and a 2 tailed test. Only age groups with adequate sample size were used for statistical comparison (20-30 years vs >50 years and 31-50 years vs >50 years). Significant p value was set up as <0.05 for all tests (14).

## Results

A total of 379 individuals consented to be a part of this study (**Supplementary Material 1**). The age of the participants was 18 years old or older. The cohort had more male donors (57%) than female (43%). The age distribution of this cohort was as follows: group 1 (<20 years old, 1%), group 2 (20-30 years old, 15%), group 3 (31-<50 years old, 44.6%) and group 4 (>50 years old, 39.3%). The entire cohort was administered two doses of the Sinopharm vaccine (**Table 1**). Notably, none of the 379 individuals that participated in this study experienced any adverse events.

**Table 1:** Age and gender demographics of the double Sinopharm Vaccinated cohort in this study.

Group	Age (years)	Female (%)	Male (%)	Total
**1**	<20	1 (0.6)	3 (1.3)	4 (1)
**2**	20-30	22 (13.3)	35 (15.7)	57 (15)
**3**	31-<50	67 (40.6)	102 (45.7)	169 (44.6)
**4**	>50	73 (44.2)	76 (34.1)	149 (39.3)
	**Total**	163 (43)	216 (57)	379 (100)

Between days 14 and 31 post double vaccination, S antibody levels were measured. We detected positive S antibody levels (>0.8 U/mL) in 364 (96%) of 379 donors. % seropositivity was slightly higher among males (205/216, 94.9%), compared to females (159/163, 92%) and was found to be highest in both females and males between 20-30 years of age (100% and 97.1%, respectively). Among donors between 31-50 years of age positive S antibody levels in females and males was 97% and 93.1%, respectively, and among those >50 years of age was 96.7% on average in both genders (**Table 2**).

**Table 2:** Quantification of SARS-CoV-2 S antibody positivity of individuals given 2 doses of Sinopharm vaccine.

Group	Age (years)	Sex	Negative		Positive		Spike antibody levels (U/mL)		
			No (%)*	No other clinical issues (%)**	No (%)*	No other clinical issues (%)**	Median	Minimum	Maximun
**1**	**<20**	**Female**	0	0	1 (100)	0	85.82	85.82	85.82
		**Male**	0	0	3 (100)	0	180.68	42.05	250
**2**	**20-30**	**Female**	0	0	22 (100)	1 (4.5)	183.97	14.97	250***
		**Male**	1 (2.9)	1 (100)	34 (97.1)	2 (5.9)	167.59	0.4	250***
**3**	**31-<50**	**Female**	2 (3)	1 (50)	65 (97)	4 (6.2)	154.54	0.41	250***
		**Male**	7 (6.9)	4 (57.1)	95 (93.1)	0	132.92	0.4	250***
**4**	**>50**	**Female**	2 (2.7)	1 (50)	71 (97.3)	11 (15.5)	98.17	0.4	250***
		**Male**	3 (3.9)	2 (66.7)	73 (96.1)	7 (9.6)	90.01	0.4	250***
** **	** **	**Total**	15 (4)	9 (60)	364 (96)	25 (6.9)	136.71	0.4	250

*Percentage of donors with negative/positive spike values of female/male in that group.

**Percentage of donors with negative/positive spike values with other clinical issues of female/male in that group.

***Some donors in this group had spike antibody levels >250 U/mL.

**Figure 1:**
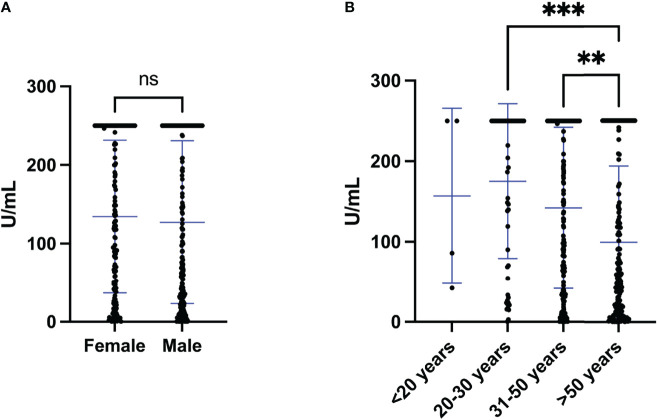
S antibody levels of individuals vaccinated with two doses of the Sinopharm vaccine. **(A)** S antibody levels in females and males after the second dose of Sinopharm vaccine. **(B)** S antibody levels in four age groups (<20, 20-30, 31-<50 and >50 years old) after the second dose of the Sinopharm vaccine. Differences between genders and groups were assessed by **(A)** Mann Whitney test and **(B)** Kruskal-Wallis test, followed by post hoc multiple comparison test. The asterisks indicate **p value < 0.01 and ***p value < 0.001. ns, not significant.

As described above, in total, 364 (96%) donors had positive S antibody levels and only 15 (4%) showed negative levels. S antibody levels were not significantly different between female and male (p=0.507) (**Figure 1A**). However, S antibody levels in population >50 years were significantly lower than either 20-30 (p value <0.001) or 31-50 (p value <0.01) year old (**Figure 1B**).

**Figure 2:**
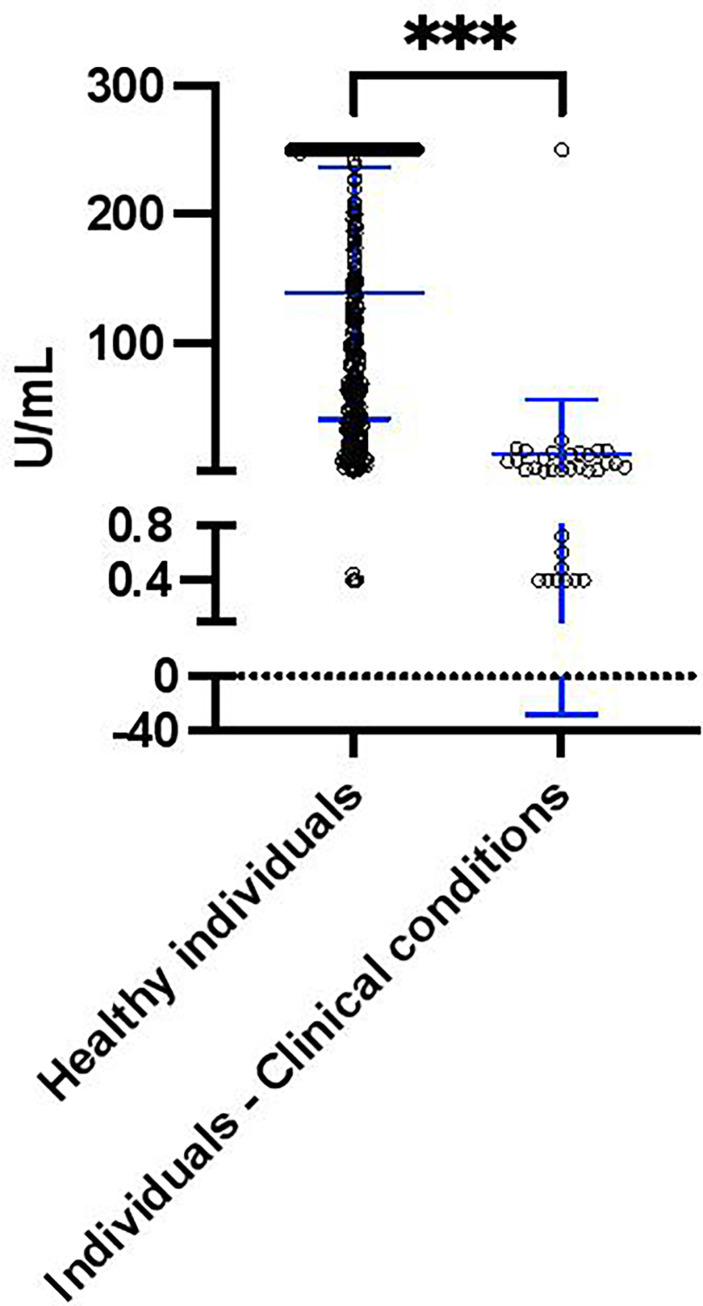
S antibody levels in healthy individuals (n = 345) and individuals with clinical conditions (including Rheumatic, Renal, Obesity, Type 2 diabetes, Psoriasis and Multiple sclerosis, n = 34) after the second dose of the Sinopharm vaccine. Differences between individuals with clinical conditions were assessed by Mann Whitney test. The asterisks indicate ***p value < 0.001.

According to clinical records, 34 of 379 (8.97%) individuals had other clinical conditions, including Rheumatic, Dyslipidemia, Thanatophoric Dysplasia type 1 and 2, Anemia, Hypothyroidism, Fatty Liver, Eczema, Hepatitis C, High Blood Pressure, Renal, Obesity, Type 2 diabetes, Psoriasis, Multiple sclerosis, Chronic Kidney, Chron’s and Renal disease. Interestingly, of the 15 individuals who had negative S antibody levels 60% (9 of 15) had other clinical conditions; whereby only 6.9% (25 of 364) of individuals with positive S antibody levels had other clinical conditions (**Table 2**). Furthermore, 7 of the 9 individuals (77.8%) with both negative S antibody levels and other medical issues were male (Rheumatic disease, Renal, Obesity, Type 2 diabetes and Multiple sclerosis) and only 2 (22.2%) were female (Psoriasis and Rheumatic disease). To determine if these clinical conditions influenced the S antibody levels of the cohort, we compared the S antibody levels of healthy individuals to those with clinical conditions. Indeed, after the second dose of Sinopharm vaccine, we found that the S antibody levels were significantly less in those with clinical conditions, when compared to those without (**Figure 2**).

**Figure 3:**
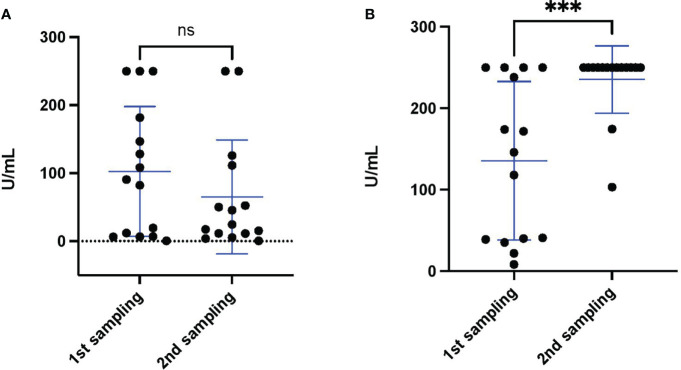
S antibody levels monitored after double vaccination with the Sinopharm vaccine and the Pfizer/BioNtech vaccine booster. **(A)** S antibody levels of 15 healthy individuals after double Sinopharm vaccination (1st sampling) and subsequent antibody level monitoring (2nd sampling). **(B)** S antibody levels of 15 healthy individuals after double Sinopharm vaccination (1st sampling) (different individuals from panel **A)** and after Pfizer/BioNtech booster (2nd sampling). Differences between groups were assessed by Mann Whitney test. The asterisk indicate ***p value < 0.001. ns, not significant.

To investigate if the S antibody levels changed over time, we analyzed the S antibody levels of 15 individuals after 14 days of vaccination with two doses of the Sinopharm vaccine (annotated “1st sampling” in the **Figure 3A**) and 16 to 119 days after the first sample collection (annotated “2nd sampling” in the **Figure 3A**) (Supplementary material 1). As is shown in **Figure 3A**, the level of S antibody after Sinopharm vaccination decreased with the time, although not with statistical significance (p value 0.34). Sinopharm double vaccinated individuals in Bahrain were recently offered a single booster vaccination (of either the Sinopharm or Pfizer/BioNtech vaccine); which was to be given at least 3 months after the second Sinopharm dose. Thus, to determine the effect of the Pfizer/BioNtech booster, we compared the S antibody levels of 15 individuals (different individuals from **Figure 3A**) after 14 days of vaccination with two doses of the Sinopharm vaccine (annotated “1st sampling” in the **Figure 3B**) and after 14 days of Pfizer booster (annotated “2nd sampling” in the **Figure 3B**). Indeed, we discovered that the S antibody levels were significantly increased after the Pfizer/BioNtech booster (**Figure 3B**).

### Strengths and Limitations

Since vaccinations were carried out in state run facilities, pre-vaccination S antibody levels were not measured, and we were unable to determine the exact time frame between final double vaccination or the booster vaccination and our blood sampling. If we had known the exact time periods for each individual, we may have been in a position to determine if shorter or longer times between vaccinations and/or our sampling correlated with S antibody levels. However, it was clear from our study that there were significant differences between our groups, even using the broader time-frame categorization. Sinopharm booster data could not be collected for this study, so we were unable to compare the effect of homologous and heterologous third doses. 15 individuals, who were part of the initial sample collection, accepted to be part of the Pfizer/BioNtech booster analysis; while this number was much lower than the initial cohort, the significant increase in S antibody levels in boosted individuals remains informative.

## Discussion

The severity of COVID-19 and the speed of SARS-CoV-2 infections nationally and internationally, have generated the most significant global health risk of our lifetimes. As a result, vaccines have been developed and approved quickly, which has already helped control the spread of SARS-CoV-2 (15). The measurement of antigen-specific antibody levels is critical in identifying vaccination efficacy and determining if different characteristics, such as age, associated clinical conditions and gender affect an individual’s responsiveness (16–18). Even though SARS-CoV-2 infection triggers antibody production against various viral antigens, antibodies against the S protein are arguably the most important, given their neutralizing activity (19). Thus, the monitoring of circulating S antibody levels is likely to give the most valuable information regarding acquired immunity against SARS-CoV-2.

In this study, we quantitated the level of antibodies directed against the S protein of SARS-CoV-2, following vaccination of different age groups with two consecutive doses of an inactivated virus vaccine (Sinopharm) in Bahrain. In addition, we measured the S antibody levels in both healthy individuals and those with clinical diseases, the majority of which are deemed “immune compromised”. The final analysis that we carried out was the effect of Pfizer/BioNtech mRNA vaccine booster upon S antibody levels of individuals who had been previously vaccinated with 2 doses of the Sinopharm vaccine. After the Sinopharm vaccination program, the level of S antibodies was found to have developed in more than 95% of the cohort. Even though gender differences have previously been reported against the SARS-CoV-2 using Pfizer/BioNTech vaccine (where females showed higher antibody titers than males) (20), we observed no statistical differences between genders. However, we found that people older than 50 years had lower levels of S antibody, when compared to the younger 31-50 year old cohort, which is in agreement with the Sinopharm report by the Strategic Advisory Group of Experts (SAGE) on immunization, from WHO^6^. SAGE reported high seropositivity in older adults, but lower geometric mean titers (GMTs), for binding antibody and neutralizing antibody, compared to younger adults. Indeed, our result was expected, given that the response to vaccines is generally reduced in older individuals, due to their immune “senescence” (21), which has been well documented for SARS-CoV-2 Pfizer/BioNTech vaccination (22). Although Sinopharm vaccines have been widely used in China, Bahrain and the United Arab Emirates, relatively few studies have evaluated antibody titres before and after Sinopharm vaccination, making our findings of significance for a large proportion of the world’s vaccination effort (23–25). Furthermore, in agreement with our findings, these previous studies similarly conclude that SARS-CoV-2 antibody production after Sinopharm vaccination was decreased with increasing age. Older people are more likely to be hospitalized or die from COVID-19 (26) and there is increased evidence that vaccination boosters help to increase antibody levels in the high-risk groups, such as older people (27).

As part of the high-risk group, individuals with comorbidities have reduced immunological responses to infection or immunization, and may thereby require higher vaccine dosages or additional booster vaccines (28). Several studies have reported that individuals with comorbidities have an increased risk of developing severe COVID-19 and subsequent death (29–31). According to the last SAGE report, due to the low number of participants, there are major gaps in our understanding of antibody levels and duration of protection against SARS-CoV-2 in people with comorbidities^5^. However, in our study, we found significantly lower S antibody levels in people with other clinical conditions. Thus, it is possible that individuals belonging to this high-risk group, would benefit from a booster vaccination, especially if their antibody levels are lower than younger or healthy groups.

Additionally, we carried out further antibody monitoring of a group of individuals who only developed low levels of S antibodies after both Sinopharm vaccine doses and found that the Pfizer/BioNTech booster significantly enhanced S antibody levels in this group. Jahromi and Al Sheikh reported, in a small study, that the Sinopharm vaccine provided protection and reduced causality, but could not prevent SARS-CoV-2 infections in Bahrain, demonstrating the value of the vaccination and suggesting that a booster program would enhance protection (25). While we were not able to compare heterologous (Pfizer/BioNTech) versus homologous (Sinopharm) COVID-19 vaccine boosters and the number of samples that we collected for Pfizer/BioNTech booster monitoring was restricted, we observed a significant increase in S antibody levels upon administration of the Pfizer/BioNTech booster vaccine. Even though homologous booster programs improve the immune response against SARS-CoV-2, two independent studies, which evaluated the effect of heterologous versus homologous COVID-19 booster vaccination, concluded that heterologous “boosting” resulted in a more robust immune response, compared to homologous “boosting” (32, 33). Going further, a comparative study in Jordanian adults, who were vaccinated with two doses of either Sinopharm or Pfizer/BioNTech vaccines, found higher immunoglobulin G in Pfizer-BioNTech vaccinated recipients (34). Other studies have also supported the value of COVID-19 boosters, especially in older groups and groups with comorbidities. Bar On et al. 2021a and Bar On et al. 2021b, confirmed that individuals older than 16, who were administered a Pfizer/BioNTech booster dose had reduced chance of infection and less severe illness, compared to those without a booster dose (35, 36). Furthermore, in agreement with our findings, others studies have also shown that individuals with comorbidities, such as Type 2 diabetes, cancer, end-stage renal disease and other immunocompromising conditions, had reduced levels of SARS-CoV-2 antibodies titers after vaccination, when compared to healthy individuals (37–40).

Lately, new mutations in the SARS-CoV-2 genome have been detected in Bahrain. Some of them have a high impact on the virulence of SARS-CoV-2 (41). Thus, the importance of efficient public health measures, viral sequencing, antibody monitoring and a continued vaccination program all remain important in our fight against this pathogen. Despite our modest sample size, our results suggest that the Pfizer/BioNtech booster vaccination program was certainly capable of generating a higher induction of S antibody levels, which suggests that a booster vaccination adds significant levels of protection, especially for those who had low levels of S antibody after the original Sinopharm double vaccination doses.

## Conclusion

Our data reveals that after two consecutive doses of Sinopharm vaccine, a high percentage of individuals (96%) develop anti-S antibodies. Although the S antibody levels following two consecutive doses of Sinopharm were positive for 96% of individuals, participants over 50 years of age and those with comorbidities, had lower levels of S antibodies, revealing that both age and the original health of an individual confound the immune antibody development. However, a third booster dose of the Pfizer/BioNtech vaccine, significantly enhanced S antibody levels, revealing a vaccination strategy that may provide enhanced protection against SARS-CoV-2.

## Data Availability Statement

The raw data supporting the conclusions of this article will be made available by the authors, without undue reservation.

## Ethics Statement

The studies involving human participants were reviewed and approved by Al Salam Specialist Hospital. The patients/participants provided their written informed consent to participate in this study.

## Author Contributions

Conceptualization, EF and NS; methodology, EF; formal analysis, JH-U; data curation, JH-U; writing—original draft preparation, JH-U; writing—review and editing, NS and JH-U; visualization, JH-U; supervision, NS; funding acquisition, EF and NS. All authors have read and agreed to the published version of the manuscript.

## Funding

This research was funded by Science Foundation Ireland, grant numbers SFI 20/SPP/3685 and SFI 19/FFP/6483.

## Conflict of Interest

The authors declare that the research was conducted in the absence of any commercial or financial relationships that could be construed as a potential conflict of interest.

## Publisher’s Note

All claims expressed in this article are solely those of the authors and do not necessarily represent those of their affiliated organizations, or those of the publisher, the editors and the reviewers. Any product that may be evaluated in this article, or claim that may be made by its manufacturer, is not guaranteed or endorsed by the publisher.

## Footnotes

1
https://covid19.trackvaccines.org/agency/who/.

2
https://www.nhra.bh/.

3
https://coronavirus.jhu.edu/region/bahrain.

4
https://www.moh.gov.bh.

5
https://epitools.ausvet.com.au/twomeanstwo.

6
https://cdn.who.int/media/docs/default-source/immunization/sage/2021/april/2_sage29apr2021_critical-evidence_sinopharm.pdf.

## Abbreviations

ACE2, Angiotensin Converting Enzyme 2; COVID-19, Corona Virus Disease; CI, Confidence interval; E, Envelope; M, Membrane; N, Nucleocapsid; Pfizer/BioNtech, Pfizer/BioNtech’s BNT162b2; RBD, Receptor binding domain; SARS-CoV-2, Severe acute respiratory syndrome coronavirus; Sinopharm, Sinopharm’s BBIBP-CorV; S, Spike; WHO, World Health Organization.
